# Emergence of a New Epidemic *Neisseria meningitidis* Serogroup A Clone in the African Meningitis Belt: High-Resolution Picture of Genomic Changes That Mediate Immune Evasion

**DOI:** 10.1128/mBio.01974-14

**Published:** 2014-10-21

**Authors:** Araceli Lamelas, Simon R. Harris, Katharina Röltgen, Jean-Pierre Dangy, Julia Hauser, Robert A. Kingsley, Thomas R. Connor, Ali Sie, Abraham Hodgson, Gordon Dougan, Julian Parkhill, Stephen D. Bentley, Gerd Pluschke

**Affiliations:** ^a^ Swiss Tropical and Public Health Institute, Basel, Switzerland; ^b^ University of Basel, Basel, Switzerland; ^c^ Wellcome Trust Sanger Institute, Wellcome Trust Genome Campus, Hinxton, Cambridge, United Kingdom; ^d^ Center de Recherche en Sante de Nouna, Nouna, Burkina Faso; ^e^ Navrongo Health Research Centre, Navrongo, Ghana; ^f^ Department of Medicine, University of Cambridge, Addenbrooke’s Hospital, Cambridge, United Kingdom

## Abstract

In the African “meningitis belt,” outbreaks of meningococcal meningitis occur in cycles, representing a model for the role of host-pathogen interactions in epidemic processes. The periodicity of the epidemics is not well understood, nor is it currently possible to predict them. In our longitudinal colonization and disease surveys, we have observed waves of clonal replacement with the same serogroup, suggesting that immunity to noncapsular antigens plays a significant role in natural herd immunity. Here, through comparative genomic analysis of 100 meningococcal isolates, we provide a high-resolution view of the evolutionary changes that occurred during clonal replacement of a hypervirulent meningococcal clone (ST-7) by a descendant clone (ST-2859). We show that the majority of genetic changes are due to homologous recombination of laterally acquired DNA, with more than 20% of these events involving acquisition of DNA from other species. Signals of adaptation to evade herd immunity were indicated by genomic hot spots of recombination. Most striking is the high frequency of changes involving the *pgl* locus, which determines the glycosylation patterns of major protein antigens. High-frequency changes were also observed for genes involved in the regulation of pilus expression and the synthesis of Maf3 adhesins, highlighting the importance of these surface features in host-pathogen interaction and immune evasion.

## IMPORTANCE

While established meningococcal capsule polysaccharide vaccines are protective through the induction of anticapsular antibodies, findings of our longitudinal studies in the African meningitis belt have indicated that immunity to noncapsular antigens plays a significant role in natural herd immunity. Our results show that meningococci evade herd immunity through the rapid homologous replacement of just a few key genomic loci that affect noncapsular cell surface components. Identification of recombination hot spots thus represents an eminent approach to gain insight into targets of protective natural immune responses. Moreover, our results highlight the role of the dynamics of the protein glycosylation repertoire in immune evasion by *Neisseria meningitidis*. These results have major implications for the design of next-generation protein-based subunit vaccines.

## INTRODUCTION

*Neisseria meningitidis* is an obligate commensal bacterium that colonizes the nasopharynx of healthy humans through transmission via respiratory secretions. For reasons not fully understood, the meningococcus can become invasive and cause disease such as septicemia or meningitis. Countries of the “meningitis belt” of sub-Saharan Africa experience the greatest burden of meningococcal disease, with incidence rates of >1,000/100,000 person-years during epidemics (1). Over the past 100 years, the meningitis belt has been affected by periodic epidemics, which occur during the dry season, stop abruptly at the onset of rain, and may flare up again in the next dry season (2). Meningococci can be divided into 12 serogroups based on the chemical composition of the capsular polysaccharide. Invasive disease is limited largely to serogroups A, B, C, W, X, and Y. Serogroup A has been responsible for the majority of the epidemics in the meningitis belt in recent years (2–4). The introduction of a monovalent A conjugate vaccine in 2011 is reducing both disease and colonization by this serogroup (5–7). A striking epidemiological feature of *N. meningitidis* in the meningitis belt is the appearance of clonal waves of colonization and disease (8). Classification of *N. meningitidis* clones is currently based primarily on multilocus sequence typing (MLST), whereby strains are given a specific sequence type (ST) number based on the presence of particular alleles of seven housekeeping genes. Serogroup A strains with the STs 5, 7, and 2859 have been responsible for most of the epidemics in the meningitis belt in the past two decades (2, 9, 10). The ST-5 clone was introduced to Africa after an epidemic in Mecca in 1987, and the ST-7 clone was found in Africa for the first time in 1995. Both clones were present in Asia (3) and Russia (3) before the introduction to Africa. In contrast, the first description of the ST-2859 clone was in Africa in 2003 (11). STs 5, 7, and 2859 are single-locus variants, i.e., they differ at only one of the seven housekeeping genes analyzed. However, MLST may dramatically underestimate genetic differences between STs, since meningococci are naturally competent for transformation by exogenous DNA (12, 13) and frequently incorporate genomic sequences acquired from co-colonizing *Neisseria* spp.

In this study, we hypothesized that herd immunity against meningococcal surface antigens is responsible for the observed disappearance of individual clones after efficient colonization of a population in the African meningitis belt for approximately 4 years. Since a rapid succession of different serogroup A STs (8, 14, 15) has been observed, colonization does not seem to be impeded primarily by natural antibody responses against the serogroup A capsular polysaccharide. Thus, immune evasion may rather be associated with changes in noncapsular antigens. To validate this hypothesis, we analyzed the genetic diversification of hypervirulent serogroup A meningococcal clones by comparative genomic analysis. Our study was focused on the microevolution between and among ST-7 and ST-2859 strains isolated between 2001 and 2009 in the course of longitudinal meningococcal colonization and disease surveys in Ghana and Burkina Faso (15, 16).

## RESULTS

### Phylogenetic and phylogeographic analysis of serogroup A ST-7 and ST-2859 *N. meningitidis* isolates.

**FIG 1:**
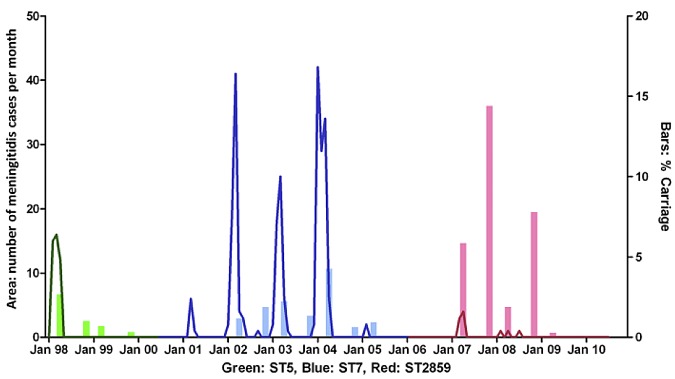
Waves of *N. meningitidis* serogroup A colonization and disease in the KND from 1998 to 2011. Serogroup A carriage rates recorded during biannual colonization surveys (April and November each year) and monthly numbers of cases of serogroup A meningococcal meningitis are depicted.

Within the framework of a longitudinal meningococcal disease and colonization study in the Kassena-Nankana District (KND) of Northern Ghana, we have observed a rapid succession of serogroup A clones (Fig. 1). After causing a major epidemic in 1997, ST-5 meningococci were still detectable in 1998 and 1999 but disappeared completely thereafter. A new colonization and disease wave, caused by serogroup A ST-7 meningococci, emerged in 2001. Again, this lineage disappeared after only a few years of colonization of the local population and was last detected in 2005. A third serogroup A colonization and disease wave associated with ST-2859 strains began in 2007. To validate the hypothesis that development of mucosal herd immunity against noncapsular antigens forms the basis for clonal succession, we performed a comparative genomic analysis with 100 serogroup A ST-7 and ST-2859 strains isolated between 2001 and 2009. The collection comprised both invasive strains isolated from cerebrospinal fluid (CSF) and carriage strains isolated from throat swabs (see Table S1 in the supplemental material). Strains isolated from two consecutive clonal waves in the neighboring KND and Bolgatanga District (BOD) of Ghana were analyzed. In addition, we analyzed ST-2859 strains from the Nouna Health District (NHD) of Burkina Faso, where this ST was first detected (2, 14).

**FIG 2:**
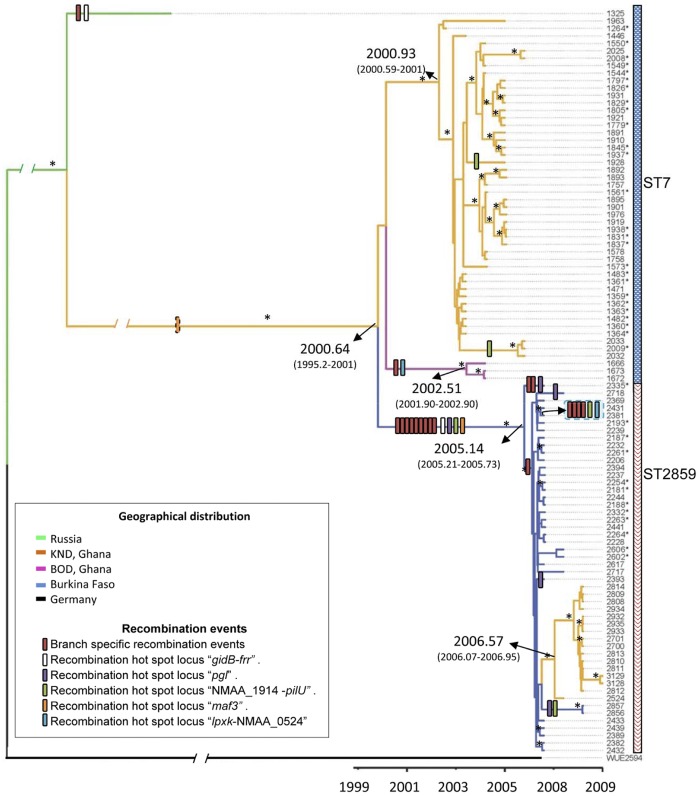
Maximum clade credibility tree from BEAST showing phylogeographic reconstruction of *N. meningitidis* strains. A phylogenetic tree of *N. meningitidis* serogroup A ST-7 and ST-2859 strains based on SNP differences across the genome, excluding predicted recombination events, is shown. The WUE2594 genome was used as an outgroup to root the tree. Branches are colored on the basis of the region of isolation of the strains. Estimates of the age of the common ancestor are indicated with arrows, which are provided as the median values, with 95% HPD given in parentheses. For these estimations, only the African strains were taken into account. Asterisks indicate Bayesian *a posteriori* probabilities of 1 for the indicated nodes. Recombination events are indicated along the branches by colored boxes. The orange box with discontinuous border lines belongs to a recombination event with uncertain location.

To establish an *N. meningitidis* phylogeny, we determined the interrelationships between the strains based on a genome-wide single-nucleotide polymorphism (SNP) analysis. We mapped the obtained sequences (average coverage of 425 reads/position) against the most closely related published genome (see Fig. S1 in the supplemental material) of the serogroup A ST-5 strain WUE2594 (17). Our sequences covered 93.8% of the complete WUE2594 reference genome. SNP calling revealed a total of 7,503 SNPs within the nonrepetitive genome (2,029,858 bp). SNPs associated with recombination blocks were removed as described previously by Croucher et al. (18), and a maximum likelihood (ML) tree was constructed using a general time reversible (GTR) evolutionary model based on the 541 SNPs not associated with recombination blocks. The tree was rooted on strain WUE2594; ST-7 and ST-2859 isolates grouped in two distinct clades (Fig. 2). A single non-African ST-7 isolate, strain 1325 from Russia, included as an outlier in the analysis, did not associate with the African ST-7 or ST-2859 clades (Fig. 2). Phylogenomic reconstruction of ST-5, ST-7, and ST-2859 isolates yielded a maximum likelihood tree that showed that ST-7 and ST-2859 isolates are more closely related to each other than to the ST-5 clade (Fig. 2). Analysis was subsequently focused on the transition from the ST-7 to the ST-2859 lineage. Within the ST-7 clade, the genomes grouped by geographical origin, with all isolates from the KND (Ghana) in one subgroup and 3 of 4 isolates from the neighboring BOD (Ghana) in another subgroup (Fig. 2). The ST-2859 strains from the NHD (Burkina Faso) and the KND (Ghana) formed two subgroups of the ST-2859 clade (Fig. 2). No separation into subgroups was observed in association with source (disease versus colonization isolates). While this analysis did not separate strains with respect to the isolation year, the older isolates tended to be in a basal position within their corresponding clades (e.g., strain 1264). The phylogeny suggests a common origin of the African ST-7 and ST-2859 isolates and a population structure influenced by geographic origin but not by the source of isolation. A phylogeographic analysis using BEAST (http://tree.bio.ed.ac.uk/software/beast/) with a Bayesian skyline model of population size change and a relaxed lognormal molecular clock produced a maximum clade credibility (MCC) tree which identified two clusters corresponding to the two sequence types (Fig. 2; see also Fig. S2 in the supplemental material), consistent with our ML phylogeny. From the BEAST analysis, we were also able to estimate the substitution rate of the African serogroup A *N. meningitidis* strains to be 3.1 × 10^−6^ substitutions per site per year (95% highest posterior density [HPD] lower value, 2.30 × 10^−6^; 95% HPD upper value, 3.85 × 10^−6^). This rate is similar to that calculated for *Staphylococcus aureus* (3 × 10^−6^) (19) and is faster than the rates estimated for *Salmonella enterica* serovar Typhimurium (1.9 × 10^−7^ and 3.9 × 10^−7^) (20) and *Yersinia pestis* (2 × 10^−8^) (21). The MCC tree indicates that the origin of the common ancestor of ST-7 and ST-2859 strains dates to 2000 (95% HPD, 1995-2001) (Fig. 2), suggesting that the ancestor of the ST-2859 strains may have emerged in Africa from ST-7 bacteria. The BEAST analysis indicates that the ST-2859 strains from Ghana are descendants of the ST-2859 lineage from Burkina Faso and that the origin of their common ancestor dates to 2005/2006, about 5 years after the divergence of the ST-7 and ST-2859 lineages (Fig. 2). The comparative genomic analysis fully confirms the MLST-based conclusion (15) of a succession of closely related hypervirulent meningococcal clones and that individual clones disappear completely after colonizing the local population for a few years.

### Analysis of the phylogenetic history of recombination blocks.

Homologous recombination is an important driving force of the adaptive evolution of *N. meningitidis* (12). To elucidate the role of recombination events in clonal succession, we investigated the recombination history of the ST-7 and ST-2859 serogroup A meningococcal populations. Thirty-four recombination blocks with high SNP density were predicted by the method of Croucher et al. (18), excluding recombination blocks detected in the WUE2594 reference genome branch and confirmed by a phylogenetic analysis (data not shown). The size of the identified recombination blocks varied between 159 bp and 11,690 bp, with a mean length of 2,465 bp. The identified recombination events are spread over the genome (Fig. 2; see also Fig. S2 in the supplemental material), affecting altogether 5.4% of the total length. The location of recombination events across the phylogenetic tree of the sequenced strains is depicted in Fig. 2. The common ancestor of all ST-2859 strains accumulated 13 recombination blocks. One recombination block is common to all African ST-7 strains (Fig. 2), and additional recombination events were identified within the ST-7 and ST-2859 clonal complexes (Fig. 2; see also Fig. S2 in the supplemental material). Most of these were shared by more than one strain, implying frequent expansion of new subclones.

Within the cluster of ST-7 strains isolated between 2001 and 2005 from northern Ghana, four strain-specific recombination blocks were identified. Two of these were shared by three strains (1666, 1672, and 1673) isolated in 2003 from carriers reporting outside the KND at the Bolgatanga District and thus being members of an emerging new local subclone. A fourth isolate, strain 1963, from this hospital did not harbor these two recombination blocks. A third ST-7 strain-associated recombination block was shared by three strains (the colonization isolates 2032 and 2033 and the CSF isolate 2009) isolated in 2005 from the same neighborhood in the KND, and a fourth recombination block was found only in one strain (1928). While no specific recombination event was found within the ST-2859 strains isolated in 2008 and 2009 in the KND of Ghana, 13 events were identified within the cluster of ST-2859 strains isolated in the NHD of Burkina Faso in 2006 and 2007 (Fig. 2; see also Fig. S2 in the supplemental material). In order to elucidate the putative origin of the recombination blocks, we performed an *a posteriori* phylogenetic analysis. While most recombination blocks identified other *N. meningitidis* strains as putative donors, eight blocks had a highest identity to published *Neisseria lactamica* genomic sequences (see Table S2 in the supplemental material).

In total, 6,962 SNPs were introduced by the 34 recombination blocks (comprising altogether 133 kb), with only 541 SNPs identified in the core genome ascribed to point mutations. A total of 3,400 of the 6,962 SNPs associated with homologous recombination events were not shared by the ST-7 and ST-2859 clades. A total of 362 of these were located in intergenic regions, 265 in pseudogenes, and 2,773 in coding regions (1,928 synonymous, 838 nonsynonymous, and seven stop mutations). There were thus 2.3 times more synonymous than nonsynonymous recombination-associated SNPs, which is much higher than the ratio (0.63) observed for putative point mutations (see below). This indicates that the alleles acquired by the recombination events were subjected to longer periods of purifying selection (22) due to sharing a much more distant common ancestor. A functional category classification (23) of the 98 genes affected by recombination revealed an overrepresentation of the “cell envelope” category (see Fig. S3 in the supplemental material). Homologous recombination thus seems to be the main evolutionary driving force for *N. meningitidis*, affecting in particular genes potentially implicated in host-pathogen interaction.

### Characterization of recombination hot spots.

**FIG 3:**
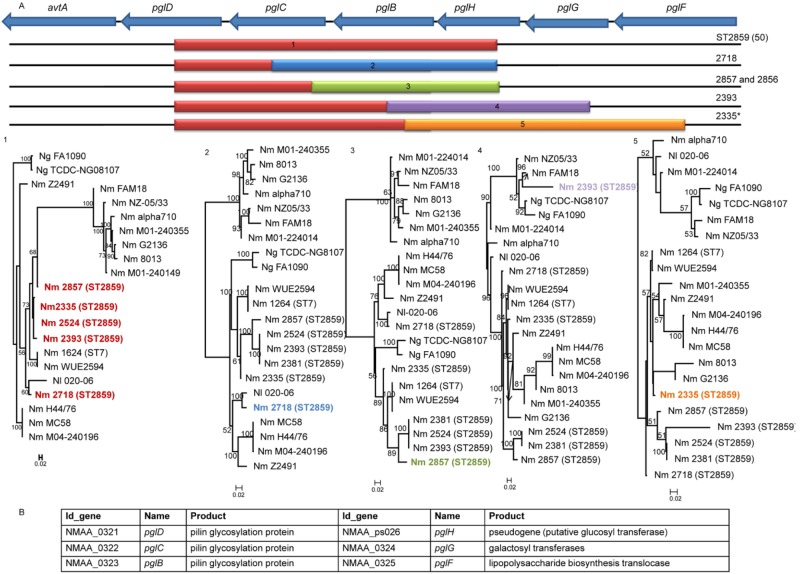
Phylogenetic reconstruction of the *pglD*-NMAA_0325 recombination hot spot. (A) The top line represents the gene content of the fragment in the reference genome, with coding sequences (CDSs) represented as blue arrows. Below the top line, the colored boxes represent recombination fragments detected in the corresponding strains. Asterisks indicate disease isolates. Maximum likelihood phylogenetic trees associated with the recombination blocks are depicted below. Support for tree nodes was assessed using 100 bootstrap replicates. (B) Table showing systematic gene identifier (ID_gene), gene name, and protein product of the genes present in the recombination hot spot. Names of the strains implicated in the recombination events at this locus are shown in color.

We identified five recombination hot spots, defined as regions of the genome associated with more than one independent recombination event (24). One of the hot spots was the pilin glycosylation protein (*pgl*) locus, which is involved in the synthesis of the *N. meningitidis* glycan repertoire (25) (Fig. 3). Compared to the ST-7 strains, the common ancestor of the ST-2859 strains has acquired a recombination block encompassing the *pglD*, *pglC*, *pglB*, and *pglH* genes, for which no closely related published sequence was identified. In addition, four other independent recombination events affecting this locus were identified in individual ST-2859 isolates (Fig. 3).

**FIG 4:**
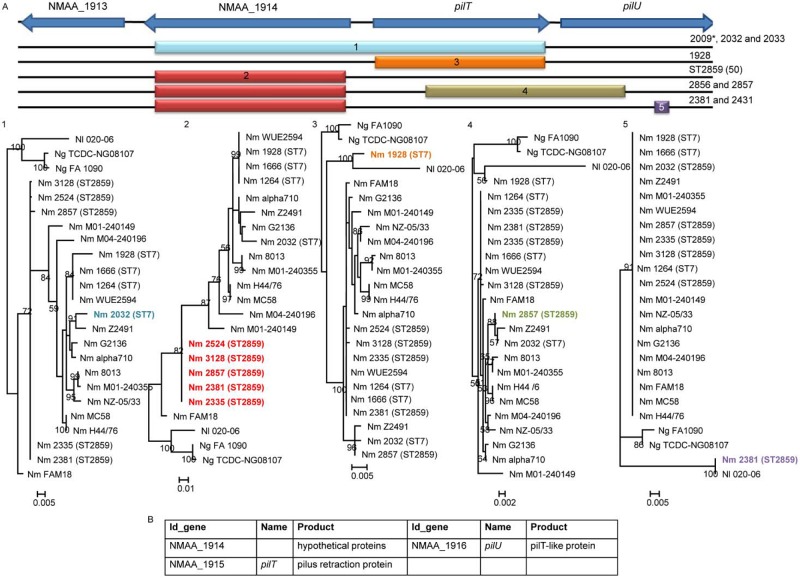
Phylogenetic reconstruction of the NMAA_1914-*pilU* recombination hot spot. (A) The top line represents the gene content of the fragment in the reference genome, with CDSs represented as blue arrows. Below the top line, the color boxes represent the recombination blocks located in the corresponding strains. Asterisks indicate disease isolates. Under the boxes, the maximum likelihood phylogenetic trees associated with the recombination blocks are depicted. Support for nodes of the trees was assessed using 100 bootstrap replicates. (B) Table showing systematic gene identifier (ID_gene), gene name, and protein product of the genes present in the recombination hot spot. Names of the strains implicated in the recombination events at this locus are shown in color.

The NMAA_1914-*pilU* locus encoding the PilU and PilT proteins, which are involved in the regulation of type IV pilus expression, was identified as another recombination hot spot (Fig. 4). At this locus, we identified a recombination block acquired by the common ancestor of the ST-2859 strains, two secondary recombination events affecting strains of the ST-2859 cluster, and two independent recombination events within strains of the ST-7 cluster.

**FIG 5:**
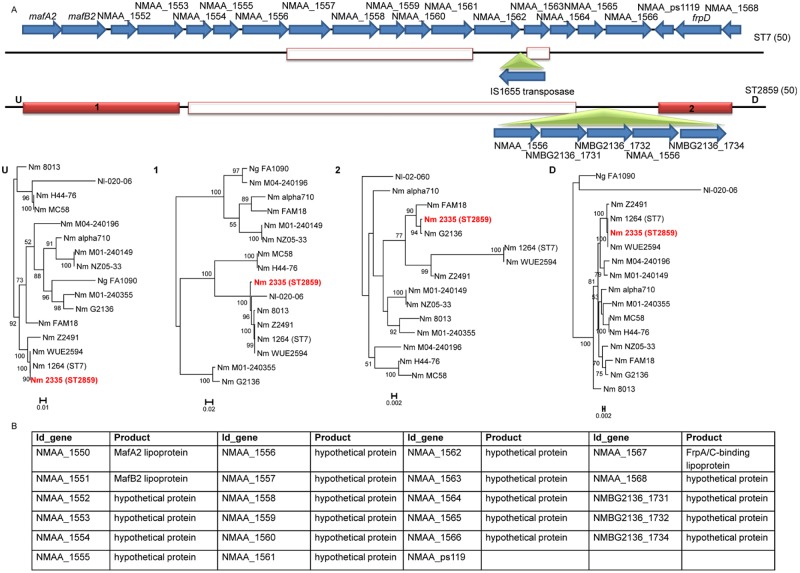
Phylogenetic reconstruction of the *maf3* recombination hot spot. (A) The top line represents the gene content of the fragment in the reference genome, with CDSs represented as blue arrows. “U” and “D” correspond to the upstream and the downstream regions. Below the top line, white boxes represent deletions and green triangles insertions. Colored boxes represent recombination blocks located in the corresponding strains. Under the boxes, the maximum likelihood phylogenetic trees associated with the recombination blocks are depicted. Support for nodes of the trees was assessed using 100 bootstrap replicates. (B) Table showing systematic gene identifier (ID_gene), gene name, and protein product of the genes present in the recombination hot spot. Names of the strains implicated in the recombination events at this locus are shown in color. We analyzed the sequences of the recombination blocks (marked red) upstream (*mafA2*-NMAA1553) and downstream (*frpD*-NMA1568) of the deletion separately.

The *maf3* locus was identified as a third recombination hot spot (Fig. 5). The tandem *mafA* and *mafB* genes in the *maf* locus are thought to encode adhesins. Compared to the WUE2594 genome, the common ancestor of the ST-7 strains carries a deletion of six genes and an insertion of the IS1655 transposase. All ST-2859 strains share a recombination block starting at *mafA2* and ending at NMAA_1568. Within this recombination block, a deletion of 11 genes and an insertion block of five genes were detected. These results suggest that the recombination event in the *maf3* locus common to all African ST-7 strains has occurred prior to the spread of this clade or the split of the African ST-7 and ST-2859 clades (Fig. 2).

Further recombination hot spots were detected for the *gidB-frr* and the *lpxK*-NMAA_0524 loci, which encompass genes with diverse functions (see Fig. S4 and S5 in the supplemental material). Taken together, at least three of the five identified recombination hot spots thus seem to be of major relevance for host-pathogen interaction.

### Analysis of point mutations.

Since we did not find a correlation between recombination events and the source of the strains (case versus carrier isolates), we tested further for association with point mutations.

**FIG 6:**
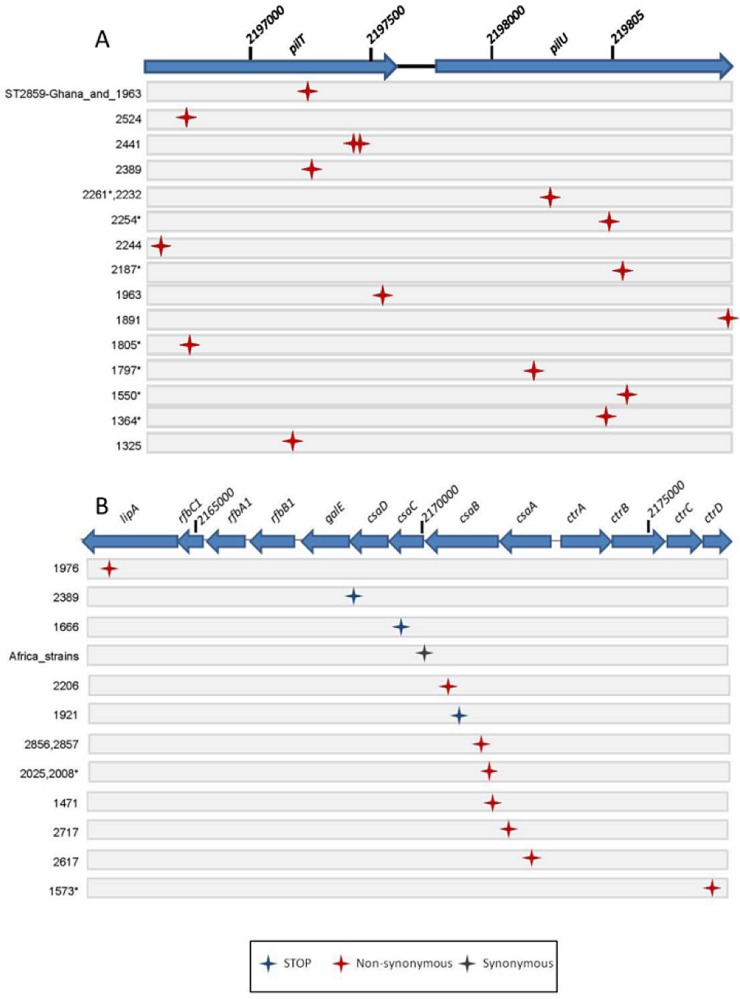
Distribution of point mutations in the two identified point mutation hot spot regions. Names of the strains carrying the respective point mutation are provided at the left side. Asterisks indicate disease isolates. The nature of the point mutation is indicated by the color (blue, stop codon; red, nonsynonymous change; gray, synonymous change). (A) The top line shows the position of the *pilT* and *pilU* genes. (B) The top line shows the position of the *lipA*, *rfbABC*, *csaABCD*, and *ctrABC* genes.

The analysis of putative point mutations revealed that only 541 (7.2%) of the 7,503 called SNPs were not associated with recombination blocks. A total of 360 (66.5%) of these putative point mutations were not shared by the ST-7 and ST-2859 clades (see Table S3 in the supplemental material). A total of 57 of these uncommon SNPs were located in intergenic regions, 23 in pseudogenes, and 280 in coding regions (91 synonymous, 176 nonsynonymous, and 13 stop mutations). Functional classification of the genes affected by point mutation revealed that genes encoding proteins of the “cell envelope” category were overrepresented (see Fig. S6 in the supplemental material). Moreover, seven times more nonsynonymous than synonymous point mutations were found in genes belonging to this category, which is indicative of positive selection (26). A one-dimensional spatial scan statistic analysis (18) of the distribution of the 360 uncommon putative point mutations identified the *lipA*-*csaABCD*-*ctrD* capsule synthesis locus (27) and the *pilT*-*pilU* locus as point mutation hot spot regions (Fig. 6; see also Fig. S7 in the supplemental material). While no correlation between the presence of point mutations and the source of strains (carriage versus disease isolates) was observed, the fact that the *pilT*-*pilU* locus is both a recombination hot spot and also frequently affected by nonsynonymous point mutations is evidence for a strong diversifying selective pressure.

## DISCUSSION

In the past 100 years, meningococcal meningitis epidemics in the African meningitis belt have been caused primarily by serogroup A strains. While in the past epidemics occurred every five to 10 years, more recently, epidemic cycles have become more irregular, which may be related to the increased mobility of the affected population. It is generally assumed that epidemic cycles are associated with the development and loss of herd immunity, and it has been suspected that natural antibody responses against the capsular polysaccharide play a key role. However, in a longitudinal meningococcal carriage and disease study in Northern Ghana, we observed a rapid succession of colonization and disease waves associated with ST-5, ST-7, and ST-2859 clonal complexes, all carrying the serogroup A capsule (15). This indicates that natural capsule serogroup-specific antibody responses do not prevent colonization of the population with a new clone shortly after colonization with another clone of the same serogroup. This led us to hypothesize that mucosal herd immunity may target primarily noncapsular antigens. Along these lines, it has been postulated before that herd immunity is selecting escape variants for particularly immunogenic proteins, such as the surface-exposed transferrin binding protein (TbpB) (28). To assess this hypothesis further, we have performed here a comparative whole-genome analysis to determine the evolutionary history and diversity of the serogroup A strains, focusing on ST-7 and ST-2859 isolates, which have succeeded each other within a short time.

Global epidemiological data indicate that the ST-7 clonal complex emerged in Asia before it was recorded in Africa for the first time in Algeria in 1995 (10). In the KND of Ghana, ST-7 caused outbreaks in the dry seasons of 2001 to 2005 (15). Worldwide, ST-2859 strains were described for the first time in 2003 in Burkina Faso (2). They have subsequently caused epidemics in several countries of the meningitis belt. However, ST-7 was not replaced by ST-2859 in all countries of the African meningitis belt. Our phylogeographic analysis is compatible with the assumption that the common ancestor of the ST-2859 strains has emerged in Africa from the ST-7 clonal complex a few years before the first description in 2003.

The history of the recombination events in the analyzed strain populations revealed that the common ancestor of all analyzed ST-2859 strains has accumulated 13 recombination blocks. The phylogenetic analysis showed that additional strain-specific recombination events occur frequently. Some of the noncommon recombination events were shared by more than one strain, demonstrating expansion of new subclones, such as three strains isolated from carriers reporting outside the KND at the nearby Bolgatanga District, which differed from all other ST-7 strains by the acquisition of two recombination blocks. While most recombination blocks exhibited high homology with sequences found in other meningococci, eight of the 35 blocks identified showed high homology with *N. lactamica* genomic sequences. *N. lactamica* is a harmless commensal and cohabits the nasopharynx with *N. meningitidis* (29), thus providing opportunity for genomic exchange.

Among the five recombination hot spots identified within the analyzed set of 100 serogroup A strains, at least three, *pgl*, NMAA_1914-*pilU*, and *maf3*, represent loci involved in host-pathogen interactions. The *pgl* locus is involved in the synthesis of the *N. meningitidis* glycan repertoire. Pili and a range of other meningococcal proteins are expressed as glycoconjugates on the bacterial cell surface. Glycan modifications of proteins may play an important role in immune evasion (25, 30) by masking conserved protein epitopes (25). Furthermore, immune responses may be directed toward the oligosaccharide structures themselves, and their variation may be highly relevant for immune evasion. Glycosylation of PilE, a variable major subunit of the pilus fiber, has been shown to affect adhesion to human epithelial cells (31). In addition, PilE also undergoes phospho-form modification (32), which seems to stimulate detachment from host cells and dissemination in the host environment (33). The competition of the phospho-form and glyco-form modifications to the same sites of occupancy may play a role in the invasion properties of *N. meningitidis* strains (34).

The NMAA_1914-*pilU* genomic region is implicated in the regulation of type IV pilus expression. While it has been shown that PilT mediates retraction of type IV pili (35) and DNA uptake (36), PilU promotes microcolony formation (37). Both proteins are required for full virulence (37).

The *maf3* locus is generally comprised of a tandem of *mafA* and *mafB* genes, both of which are thought to encode adhesins involved in tight adherence (23). MafA and MafB are upregulated during culture in human blood, suggesting that they may also be involved in the interaction with blood cells or the survival in the bloodstream (38).

A distribution analysis of the putative point mutations, comprising about 7% of the called SNPs, revealed two mutation hot spot regions: the *pilU*-*pilT* locus, which is also a recombination hot spot, and the *lipA*-*csaABCD*-*ctrD* locus involved in capsule production. While the capsule plays an important role in resistance to complement killing and phagocytosis, it may also hinder adhesion (39). The point mutations in the *csaA* and *csaB* genes in some of the strains may influence capsule polysaccharide amount and structure. On the other hand, the observed mutations in *csaB*, *csaC*, and *csaD* leading to stop codons and production of truncated proteins may play a role in the formation of biofilms, because unencapsulated meningococcal cells bind to epithelial cell surfaces much more efficiently than encapsulated meningococci (40, 41). In the course of our longitudinal colonization study, we have regularly observed capsule-deficient variants of the dominating clones (8).

Taken together, our data indicate that the main mechanism leading to the emergence of new *N. meningitidis* clones is homologous recombination resulting in complex changes in the antigenic makeup of the bacterial cell surface. The identified genomic hot spots of recombination and point mutation are potentially relevant for both immune evasion (25, 30, 33, 37, 42) and biofilm formation (43) and can thus influence colonization and transmission frequency defining the fitness of a strain (43).

It is generally assumed that meningococci belonging to a virulent clone may cause invasive disease in some of the colonized individuals by translocating across the nasopharyngeal epithelium and multiplying in the bloodstream. Translocation across the blood-brain barrier may in turn lead to the development of meningitis and/or septicemia. Here, we observed no common genomic differences, such as point mutations in particular loci (44), between colonization and disease isolates. This indicates that phenotypic changes rather than the expansion of genetic variants in the bloodstream play a key role in the switch from asymptomatic colonization to invasive disease. Phase variable genes not analyzed in detail in the present study may play a key role in the adaptation from the carriage to the invasive state.

Acquisition of just 13 recombination blocks allowed the ST-2859 bacteria to colonize human populations which had shortly before suffered an ST-7 outbreak. Identification of recombination hot spots and analysis of the phenotype of the observed genomic changes represent an eminent approach to gain insight into targets of protective natural immune responses and immune evasion mechanisms. Our results thus have major implications for the design of next-generation protein-based subunit vaccines.

## MATERIALS AND METHODS

### Preparation and sequencing of genomic DNA.

*N. meningitidis* strains (see Table S1 in the supplemental material) were grown in liquid brain heart infusion (BactoTM) medium, and chromosomal DNA was prepared as described (45, 46). Briefly, bacterial pellets were resuspended in 0.5 ml of TES buffer (50 mM Tris, 20 mM EDTA, 50 mM NaCl [pH 8]). A total of 2 µl RNase A (Qiagen 100 mg/ml) and 20% SDS were added to a final concentration of 1%, and cells were lysed for 5 min at 42°C. After two phenol-chloroform-isoamyl alcohol (25:24:1; Sigma) extractions and one chloroform-isoamyl alcohol (24:1; Sigma) extraction, DNA was precipitated in two volumes of isopropanol followed by suspension in TE buffer (10 mM Tris, 1 mM EDTA [pH 8]). Ammonium acetate was added to a final concentration of 2.5 M, and the DNA was precipitated in two volumes of ethanol. After two 70% ethanol wash steps and subsequent drying, the DNA was diluted in TE buffer (10 mM Tris, 0.1 mM EDTA [pH 8]).

Multiplexed genomic DNA libraries were prepared with an insert size of 200 using 24 unique index tags. Libraries were combined into pools of 24 and sequenced on an Illumina HiSeq for 75 cycles from each end to produce paired-end reads plus an 8-base index sequence read. Downstream analysis used the index tags to assign reads to individual samples.

### PCR-based analysis of the *maf3* locus sequence.

PCRs were performed on genomic DNA to confirm the *maf3* locus sequence. PCR was performed in 15-µl reaction mixtures containing 0.5-ng genomic DNA, 5 µl of 5× Q solution (Qiagen), 1.5 μl 10× reaction buffer (Qiagen), 0.3 µl 25 mM MgCl_2_, 0.3 µl 10 mM deoxynucleoside triphosphates (dNTPs) (Sigma), 0.12 µl TopTaq polymerase (Qiagen), and 5 µM of each primer (NMA2112F, 5′ TCCAGCTTACGGAAAAAGAATCC 3′; NMA2112R, GGAAAACCTATGGGATGATACGG 3′). PCR conditions were 94°C for 2 min, 30 cycles of 94°C for 20 s, 53°C for 45 s, and 72°C for 3 min, and finally 72°C for 10 min. PCR products were sequenced using the primers used for PCR amplification and additional sequencing primers (SeqNMA2112F, 5′ GGACATCGTGATTGGAATCG 3′; SeqNMA2112R, 5′ AAAGCATTCGGATTTTCAGG 3′; SeqNMA2112, 5′ CCAAAACAGCCTACGTCTTGC 3′).

### Read alignment and SNP detection.

Variation in the form of single nucleotide polymorphisms (SNPs) was detected using a mapping approach. The paired-end Illumina reads were mapped against the *N. meningitidis* serogroup A ST-5 strain WU2594 as the reference (accession number FR774048) with an insert size between 50 and 400 bp using SMALT (http://www.sanger.ac.uk/resources/software/smalt/). SNPs were identified using SAMtools (47) as previously described (18). SNPs called in phage sequences and repetitive regions of the *N. meningitidis* reference genome were excluded. Repetitive regions were defined as exact repetitive sequences of ≥50 bp in length, identified using repeat-match (48). If 10% of the genomes had an indetermination in a called SNP, these positions were removed from the analysis.

### Identification of recombination events.

Ancestral sequences were reconstructed onto each node of the phylogeny using PAML (49). From these ancestral sequences, SNPs were reconstructed onto branches of the tree. To identify recombination events, we applied a moving window approach similar to that previously described (18). All strains with possible recombination fragments were assembled using Velvet v1.0.12 (50) *de novo* assemblies with contigs realigned by Abacas (51). Draft genomes were used to query the WUE2594 genome using BLASTN, and comparison files were generated and viewed using the Artemis comparison tool (ACT) (52). To confirm the recombination fragments and to identify the most probable donor of the fragment, the predicted recombination fragments and their upstream and downstream flanking regions were extracted from the assembled genomes and aligned with their homologues from 12 publically available *N. meningitidis* (Z2491 [AL157959.1], WUE2594 [FR774048.1], M01-240355 [CP002422.1], M01-240149 [CP002421.1], M04-240196 [CP002423.1], NZ05/33 [CP002424.1], alpha710 [CP001561.1], H44/76 [CP002420.1], MC58 [AE002098.2], G2136 [CP002419.1], FAM18 [AM421808.1], 8013 [FM999788.1]), two *N. gonorrhea* (FA1090 [AE004969.1] and TCDC-NG08107 [CP002440.1]), and one *N. lactamica* (020-06, [FN995097.1]) genomes. The alignments were used to reconstruct maximum-likelihood phylogenetic trees with RAxML v7.0.4 (53) using a general time-reversible (GTR) substitution model with γ correction for among-site rate variation. Support for nodes on the trees was assessed using 100 bootstrap replicates. We considered a recombination fragment confirmed when the cluster differed from the clusters belonging to the upstream and downstream flanking regions. Recombination hot spots are defined as regions in a genome that exhibit elevated rates of recombination, relative to a neutral expectation (24, 54).

### Phylogenetic analyses.

The network-like relationship of strains was determined using the NeighborNet algorithm as implemented in SplitsTree v4 using the multiple-sequence alignment of the concatenated MLST genes.

A maximum-likelihood phylogenetic tree was generated from the whole-genome sequence alignment without SNPs associated with recombination events. Phylogenetic trees were constructed as described above.

To estimate the mutation rate and ages of the phylogenetic nodes, Bayesian inference was implemented in BEAST (55). BEAST was run on the African strain alignments using a relaxed molecular clock, a skyline model of effective population size, and a general time-reversible evolutionary model with gamma correction. Two independent chains of 100 million steps were run, and each was sampled every 10,000 steps to ensure good mixing. The first 10 million steps of each chain were discarded as a burn-in. The results were combined using Log Combiner, and the maximum clade credibility tree was generated using Tree Annotator, both parts of the BEAST package (http://tree.bio.ed.ac.uk/software/beast/). Convergence of the two chains and the effective sample size values of estimated parameters were checked using Tracer 1.5 (available from http://tree.bio.ed.ac.uk/software/tracer). ESS values in excess of 200 were obtained for all parameters.

### Statistical testing of point mutation hot spot regions.

To identify regions of the genome which are hot spots for point mutations, we compared the reference genome with a pseudogenome containing all of the SNPs identified in at least one isolate in the study, excluding those identified as being introduced by homologous recombination. We then applied a spatial scanning statistic, as described previously in reference 18, to identify regions of the genome containing significantly higher mutation densities than would be expected by chance.

## SUPPLEMENTAL MATERIAL

Figure S1 NeighborNet tree of meningococci and other *Neisseria* spp. based on MLST. Download Figure S1, PDF file, 0.04 MB

Figure S2 Reconstruction of recombination events on non-homoplasic sites within the phylogeny of the studied serogroup A ST7 and ST2859 meningococci. Download Figure S2, PDF file, 0.1 MB

Figure S3 Functional categories of proteins affected by homologous recombination. Download Figure S3, PDF file, 0.03 MB

Figure S4 Phylogenetic reconstruction of “*gidB-frr*” hot spot recombination in *Neisseria* strains. Download Figure S4, PDF file, 0.2 MB

Figure S5 Phylogenetic reconstruction of “*lpxK*-NMAA_0524” hot spot recombination in *Neisseria* strains. Download Figure S5, PDF file, 0.2 MB

Figure S6 Functional categories of proteins affected by point mutations. Download Figure S6, PDF file, 0.07 MB

Figure S7 Distribution of point mutations along the genome in discrete windows of 6,060 bp. Download Figure S7, PDF file, 0.1 MB

Table S1 Mapping statistics and metadata for the bacterial isolates used in the study. Table S1, XLS file, 0.04 MB.

Table S2 Recombination blocks detected in the strain collection. Table S2, XLS file, 0.03 MB.

Table S3 Point mutations detected in the strain collection. Table S3, XLS file, 0.07 MB.
