# Supplementary material for: Emergence of a New Epidemic Neisseria meningitidis Serogroup A Clone in the African Meningitis Belt: High-Resolution Picture of Genomic Changes That Mediate Immune Evasion
Source: mBio. 2014 Oct 21;5(5):e01974-14. doi: 10.1128/mBio.01974-14 (PMC4212839; doi:10.1128/mBio.01974-14)
Supplement: Figure S1 — NeighborNet tree of meningococci and other Neisseria spp. based on MLST. Download [file mbo005142031sf01.pdf]

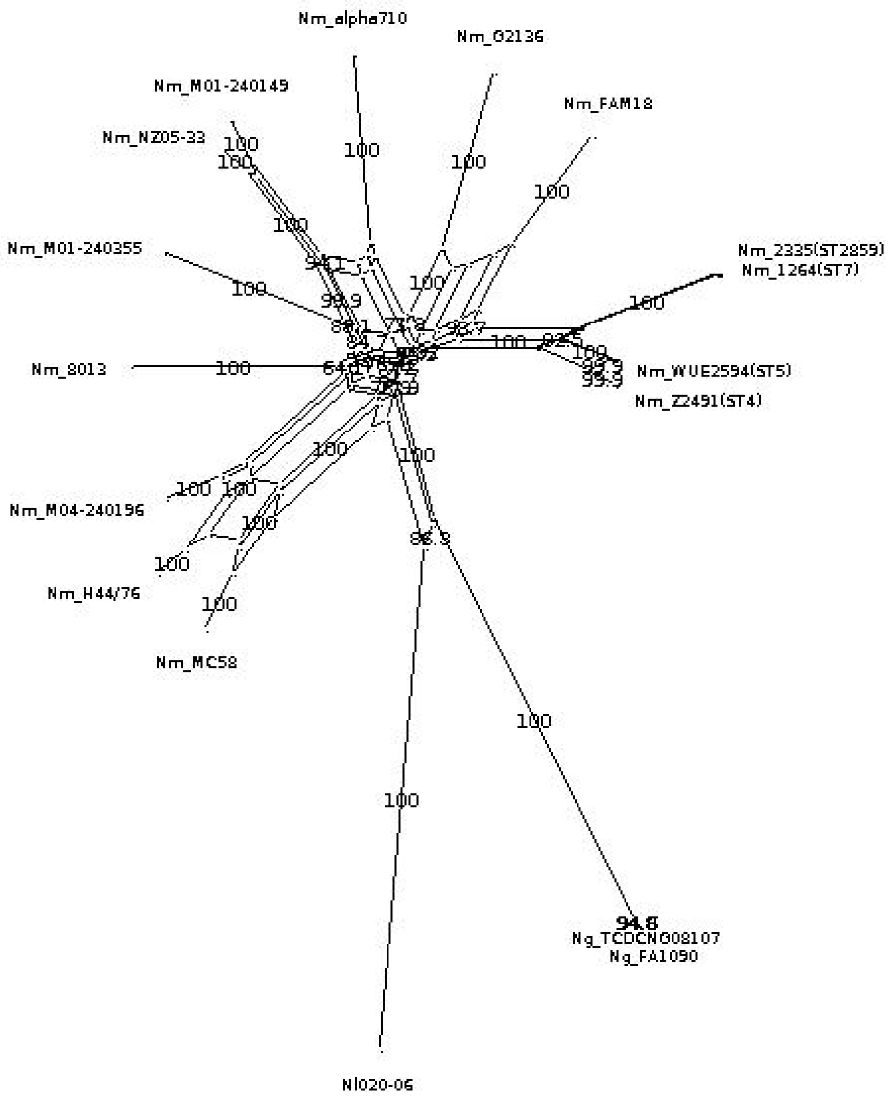

Figure S1. NeighborNet tree of meningococci and other *Neisseria* spp. based on MLST. Nm*: Neisseria meningitidis,* Nl: *Neisseria lactamica,* Ng: *Neisseria gonorrhoeae.* Bootstrap values are labeled along branches.
