# Supplementary material for: Emergence of a New Epidemic Neisseria meningitidis Serogroup A Clone in the African Meningitis Belt: High-Resolution Picture of Genomic Changes That Mediate Immune Evasion
Source: mBio. 2014 Oct 21;5(5):e01974-14. doi: 10.1128/mBio.01974-14 (PMC4212839; doi:10.1128/mBio.01974-14)
Supplement: Figure S2 — Reconstruction of recombination events on non-homoplasic sites within the phylogeny of the studied serogroup A ST7 and ST2859 meningococci. Download [file mbo005142031sf02.pdf]

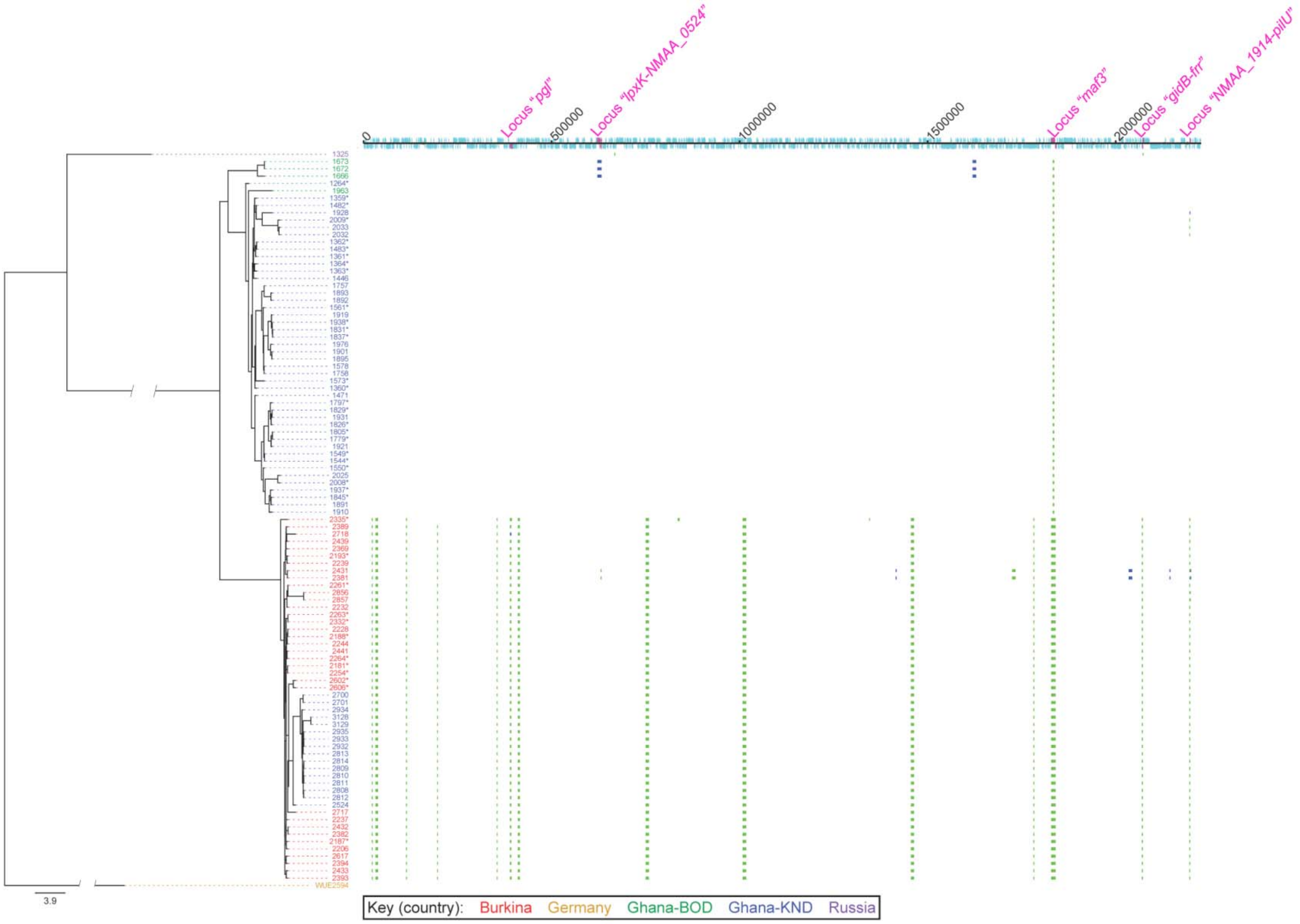

Figure S2. Reconstruction of recombination events on non-homoplasic sites within the phylogeny of the studied serogroup A ST7 and ST2859 meningococci. The top line represents the full chromosome of *N. meningitidis* strain WUE2594, with coding sequences represented as blue boxes on the relevant coding strand. The pink boxes represent recombination hot spot loci with the names of the corresponding loci written next to them. Each horizontal track represents the chromosome of a strain aligned with the phylogeny on the left. Blocks shown on the tracks represent the location of recombination fragments received by homologous replacement, with their color corresponding to the color of the most probable donor (green: *N. meningitis* and blue: *N. lactamica).*
