# Supplementary figures and images for: Emergence of a New Epidemic Neisseria meningitidis Serogroup A Clone in the African Meningitis Belt: High-Resolution Picture of Genomic Changes That Mediate Immune Evasion
Source: mBio. 2014 Oct 21;5(5):e01974-14. doi: 10.1128/mBio.01974-14 (PMC4212839; doi:10.1128/mBio.01974-14)

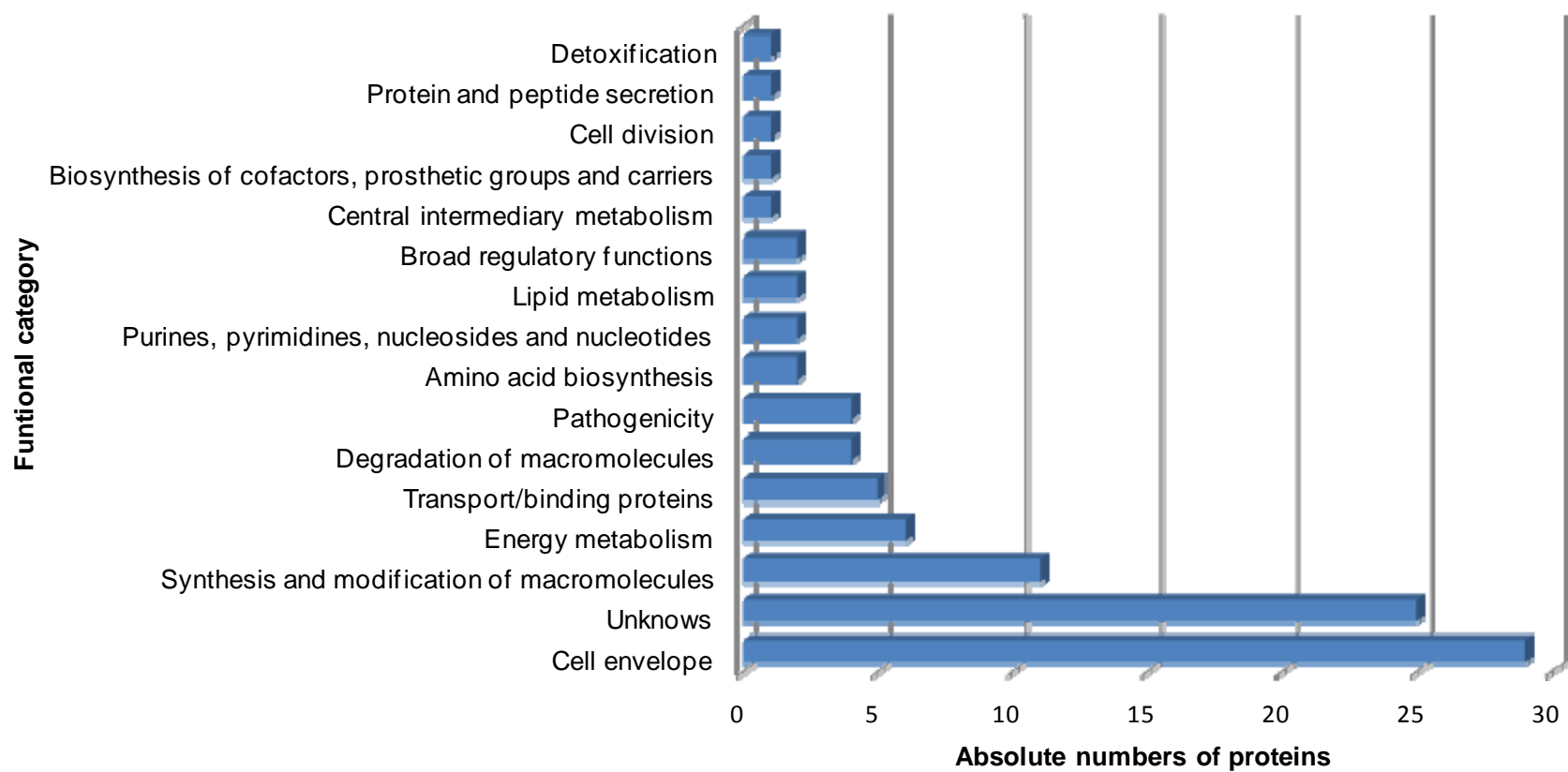

**Figure S3. Functional categories of proteins affected by homologous recombination.**

Supplement: Figure S3 — Functional categories of proteins affected by homologous recombination. Download [file mbo005142031sf03.pdf]
