# Supplementary material for: Emergence of a New Epidemic Neisseria meningitidis Serogroup A Clone in the African Meningitis Belt: High-Resolution Picture of Genomic Changes That Mediate Immune Evasion
Source: mBio. 2014 Oct 21;5(5):e01974-14. doi: 10.1128/mBio.01974-14 (PMC4212839; doi:10.1128/mBio.01974-14)
Supplement: Figure S4 — Phylogenetic reconstruction of “gidB-frr” hot spot recombination in Neisseria strains. Download [file mbo005142031sf04.pdf]

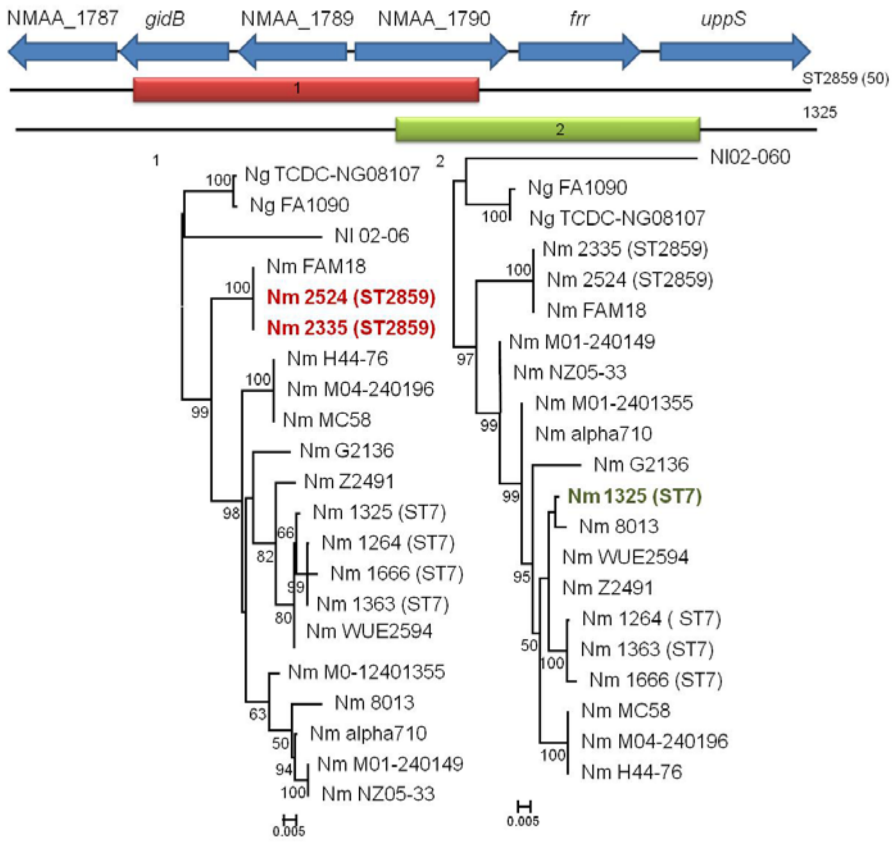

Id_gene	Name	Product	Id_gene	Name	Product
NMAA_1788	*gidB*	methyltransferase GidB (glucose-inhibited division protein B) (EC:2.1.-.-)	NMAA_1791	*frr*	ribosome recycling factor
NMAA_1789		hypothetical protein	NMAA_1792	*uppS*	undecaprenyl pyrophosphate synthetase
NMAA_1790		Inner membrane protein yeeA			

Figure S4. Phylogenetic reconstruction of "*gidB-frr*" hot spot recombination in *Neisseria* strains. **(a)** The top line reflects the gene content of the fragment, with CDSs represented as blue arrows. Below the top line the colored boxes symbolize the recombination region locate in the correspondent strains. Under the boxes are the maximum likelihood phylogenetic trees belong to recombination fragment. The trees were performed using a general time-reversible (GTR) substitution model with γ correction for among-site rate variation. Support for nodes on the trees was assessed using 100 bootstrap replicates. (**b)** Table with the, Id , name and product of the genes present in the fragment. The strains implicated in the recombination are depicted in color. The first event affected *gidB*, NMAA_1789, and NMAA_1790. The sequence stretch found in the ST2859 strains has 100% identity with the corresponding sequence of the *N. meningitidis* serogroup C strain FAM18. The second event, in the Russian ST7 isolate, affected in addition to the NMAA_1790, *frr* and *UppS*.
