# Supplementary material for: Emergence of a New Epidemic Neisseria meningitidis Serogroup A Clone in the African Meningitis Belt: High-Resolution Picture of Genomic Changes That Mediate Immune Evasion
Source: mBio. 2014 Oct 21;5(5):e01974-14. doi: 10.1128/mBio.01974-14 (PMC4212839; doi:10.1128/mBio.01974-14)
Supplement: Figure S5 — Phylogenetic reconstruction of “lpxK-NMAA_0524” hot spot recombination in Neisseria strains. Download [file mbo005142031sf05.pdf]

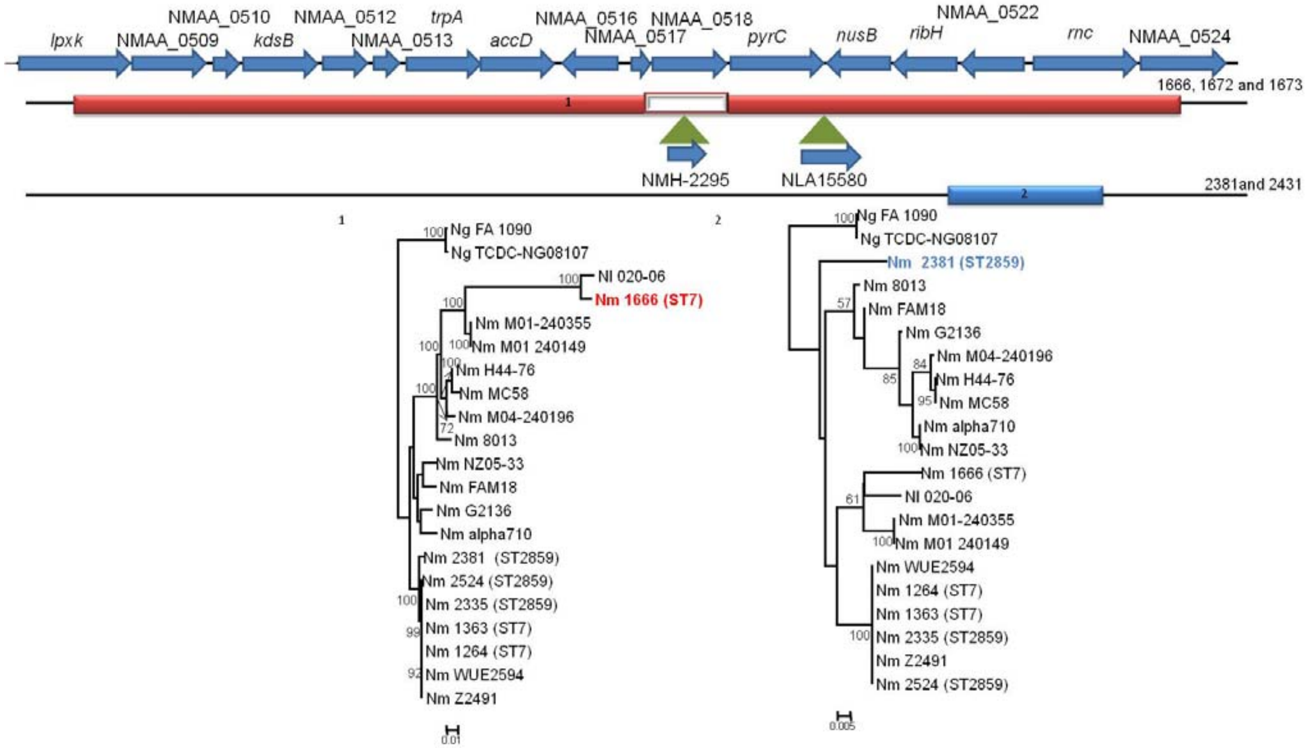

Id_gene	Name	Protein	Id_gene	Name	Protein
**NMAA_0508**	***lpxK***	Tetraacyldisaccharide-1-P 4'-kinase	**NMAA_0518**		Hypothetical lipoprotein
**NMAA_0509**		Hypothetical protein	**NMAA_0519**	***pyrC***	Dihydroorotase (3.5.2.3)
**NMAA_0510**		Hypothetical protein	**NMAA_0520**	***nusB***	RNA polymerase antitermination factor
**NMAA_0511**	***kdsB***	3-deoxy-manno-octulosonate cytidylyltransferase	**NMAA_0521**	***ribH***	6,7-dimethyl-8-ribityllumazine synthase
**NMAA_0512**		Hypothetical protein	**NMAA_0522**		Hypothetical protein
**NMAA_0513**		Hypothetical protein	**NMAA_0523**	***rnC***	Ribonuclease III (EC 3.1.26.3)
**NMAA_0514**	***trpA***	Tryptophan synthase alpha chain	**NMAA_0524**		Putative Era-like GTP-binding protein
**NMAA_0515**	***accD***	Cetyl-CoA carboxylase transferase beta subunit	**NMH-2295**		Lipoprotein
**NMAA_0516**		Conserved hypothetical periplasmic protein	**NLA15580**		Hypothetical protein
**NMAA_0517**		Putative SirA-like protein			

Figure S5. Phylogenetic reconstruction of "*lpxK-NMAA_0524*" hot spot recombination in *Neisseria* strains. **(a)** The top line reflects the gene content of the fragment, with CDSs represented as blue arrows. Below the top line the colored boxes symbolize the recombination region locate in the correspondent strains. Below the top line the white boxes correspond to a deletion and the green triangle an insertion locate in the correspondents strains. Under the boxes are the maximum likelihood phylogenetic trees belong to recombination fragment. The trees were performed using a general time-reversible (GTR) substitution model with γ correction for among-site rate variation. Support for nodes on the trees was assessed using 100 bootstrap replicates. (**b)** Table with the, Id , name and product of the genes present in the fragment. The strains implicated in the recombination are depicted in color.
