# Supplementary material for: Emergence of a New Epidemic Neisseria meningitidis Serogroup A Clone in the African Meningitis Belt: High-Resolution Picture of Genomic Changes That Mediate Immune Evasion
Source: mBio. 2014 Oct 21;5(5):e01974-14. doi: 10.1128/mBio.01974-14 (PMC4212839; doi:10.1128/mBio.01974-14)
Supplement: Figure S6 — Functional categories of proteins affected by point mutations. Download [file mbo005142031sf06.pdf]

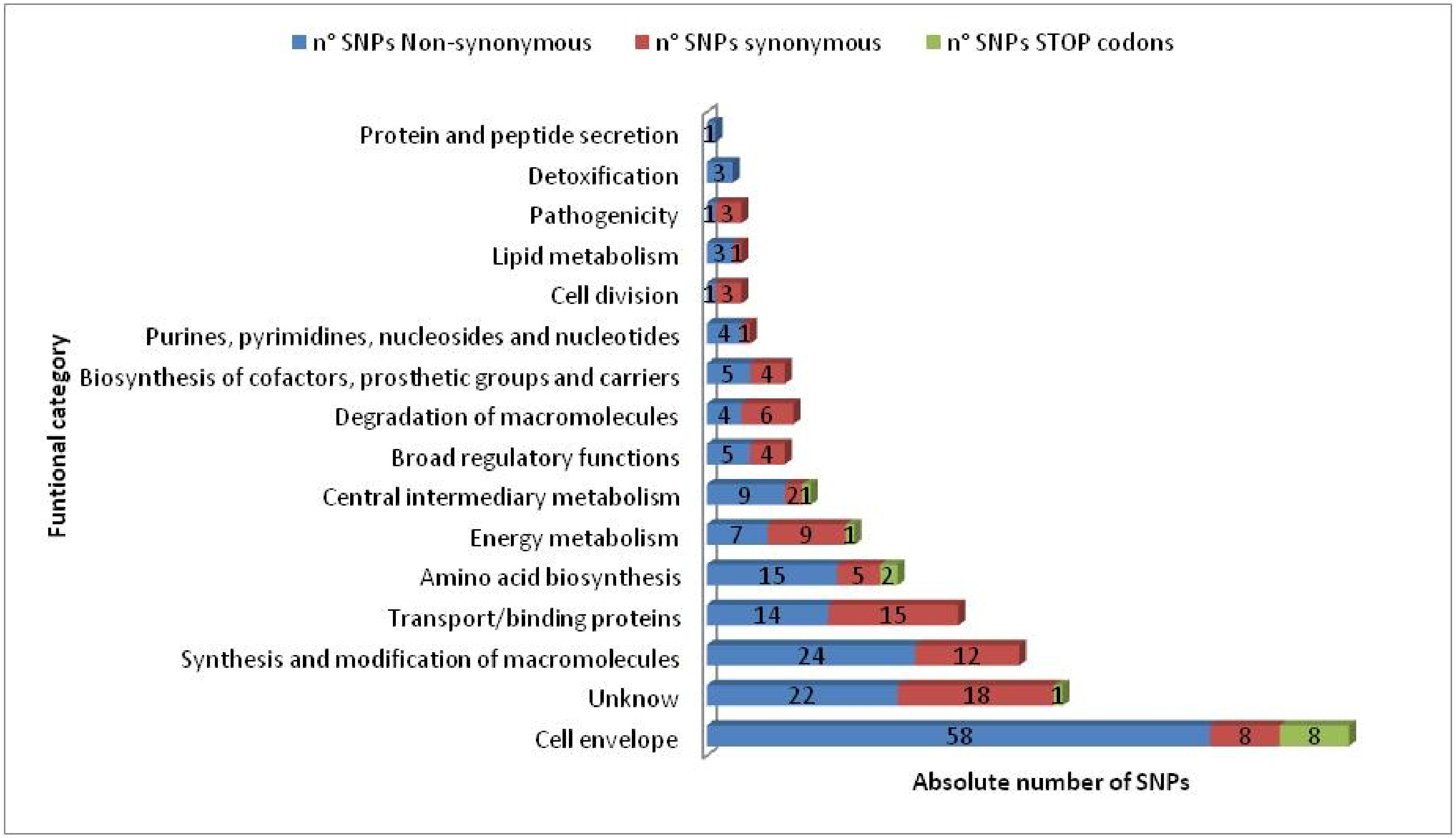

Figure S6. Functional categories of proteins affected by point mutations. Blue bars represent non-synonymous SNPs, red bars synonymous SNPs, green bars SNPs that produce a STOP codon.
